# The Effect of MWCNTs Filler on the Absorbing Properties of OPEFB/PLA Composites Using Microstrip Line at Microwave Frequency

**DOI:** 10.3390/ma13204581

**Published:** 2020-10-14

**Authors:** Ismail Ibrahim Lakin, Zulkifly Abbas, Rabaah Syahidah Azis, Nor Azowa Ibrahim, Mohd Amiruddin Abd Rahman

**Affiliations:** 1 Department of Physics, Faculty of Science, University Putra Malaysia, Serdang 43400 UPM, Selangor, Malaysia; gs51771@student.upm.edu.my (I.I.L.); rabaah@upm.edu.my (R.S.A.); mohdamir@upm.edu.my (M.A.A.R.); 2 Institute of Advanced Materials, Universiti Putra Malaysia, Serdang 43400 UPM, Selangor, Malaysia; 3 School of Graduate Studies, Universiti Putra Malaysia, Serdang 43400 UPM, Selangor, Malaysia; norazowa@upm.edu.my

**Keywords:** polymer, multi-walled carbon nanotubes, dielectric properties, S-parameters, electromagnetic waves absorption

## Abstract

Oil palm empty fruit bunch (OPEFB) fiber/polylactic acid (PLA)-based composites filled with 6–22 wt.% multi-walled carbon nanotubes (MWCNTs) were prepared using a melt blend method. The composites were analyzed using X-ray diffraction (XRD), Fourier transforms infrared (FTIR), field emission scanning electron microscopy (FESEM), and transmission electron microscopy (TEM) of the MWCNTs. The composites were characterized for complex permittivity using the coaxial probe at 8–12 GHz range and the transmission/reflection coefficients were measured through micro strip line. The dielectric permittivity measurements carried out at X-band frequency revealed that 22 wt.% MWCNTs nanocomposite display higher dielectric constant ($\varepsilon^{\prime }$) and dielectric loss ($\varepsilon^{\prime \prime }$) values of 4.23 and 0.65, respectively. A maximum absorption loss of 15.2 dB was obtained for the 22 wt.% nanocomposites at 11.75 GHz. This result suggests that PLA/OPEFB/MWCNTs composites are a promising cheap and lightweight material for the effective microwave absorption in the X-band frequency range.

## 1. Introduction

The shielding and absorption of electromagnetic (EM) waves from interference continually attract significant interest in both defense and industrial purposes. Diverse materials, whether dielectric or magnetic, were investigated for its use and application as radar-absorbing materials (RAMs) [1,2]. RAMs are being used to modify electromagnetic radiation by absorption and heat dissipation and some supporting environmental safety applications, darkroom microwaves, radar stealth, etc. [3,4]. A good EM radiation absorbing material should possess low weight, low thickness, low cost, high mechanical resistance, durability, and microwave absorption properties over a wide frequency range [5]. In this aspect, an electrically conductive composite polymer matrix is widely studied as an effective RAM. Microwave absorbers undergo dielectric and magnetic losses depending on the filler type used. However, the magnetic absorbers have some disadvantages, such as thinner matching thickness, higher weight, weaker high-frequency absorption characteristics compared with dielectric ones [6,7]. Carbonaceous solids which may be either synthetic or natural have received considerable interest in microwave absorber development due to their outstanding electrical properties and can be used in both electromagnetic interference (EMI) shielding and absorption [8]. Multi-walled carbon nanotubes (MWCNTs) were given considerable attention because of their superior mechanical properties and their high electrical and thermal conductivity. Its ability to form a proper network within the composite can enhance conductivity; thus, absorption performance will be improved by incorporation of MWCNTs [9]. Biodegradable polylactic acid (PLA)/multi-walled carbon nanotube (MWCNT) composites for high-performance electromagnetic shielding interference (EMI) application at X-band frequency was reported [10]. In another study, the effect of the polypyrrole/MWCNT content on microwave shielding efficiency was investigated. Total shielding effectiveness dominated by absorption up to −46 dB was attained in the X-band frequency range [11].

Abdallahadi et al. [12] recently reported that compacted oil palm empty fruit bunch (OPEFB) fiber of 200 µm had a high dielectric loss in the 8–12 GHz range. Malaysian oil palm mills generate approximately 15 million tons of OPEFB fiber annually [13] which is characterized by low density and good thermal and mechanical properties. OPEFB reinforced materials could reduce costs, enhance dielectric properties, thermal stability, and increase stiffness [14]. PLA is a thermoplastic polyester flexible and obtained mostly from materials that are renewable annually. PLA has high strength, elasticity modulus, stiffness, fragility, and is a biodegradable matrix [12,15]. Reinforcing thermoplastics with cellulosic fibers, however, considerably increases rigidity and strength while drastically reducing composite toughness [16]. In [17], the possibility of the application of graphite-polylactic acid (G/PLA) composite for absorption and shielding is reported. Metal-plastic composites are normally heavy and need a low print speed to produce good quality. The G/PLA composite, on the other hand, is lightweight with print speed and similar settings to standard PLA filament. PLA loaded with carbon nanotubes, graphite nanoplatelets, and other nanocarbons are among the best candidates for the production of structures with various sophisticated geometries through additive manufacturing [18].

In this study, the dielectric properties and absorption characteristics of OPEFB/PLA/MWCNTs nanocomposites were discussed. A total of 6–22 wt.% of MWCNTs filler loads were added into the OPEFB/PLA matrix to fabricate composites via melt-blend technique and characterized at 8–12 GHz range. COMSOL Multiphysics, finite element method (FEM) was used to calculate numerically the transmission coefficients of the samples using the geometry of the microstrip model. The electric field distributions due to material absorption were also examined. The main aim of this study was to examine the impact of various MWCNTs loadings on dielectric permittivity and absorption characteristics of the OPEFB/PLA/MWCNTs nanocomposite materials.

## 2. Experimental

### 2.1. OPEFB/PLA/MWCNTs Composites Preparation

**Figure 1:**
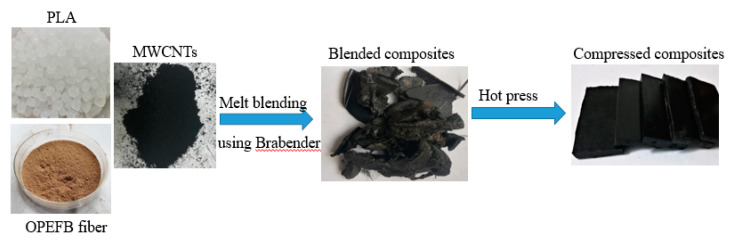
Fabrication of OPEFB/PLA/MWCNTs composites.

The OPEFB fiber was obtained from Hulu Langat Oil Palm Mill, Dengkil Selangor, Malaysia. To remove the wax layer of fibers, the OPEFB fibers were soaked in distilled water for 24 h. The fibers were rinsed with acetone and dried in an oven at 80 °C for 6 h to reduce the moisture. The dried fibers were crushed into powder using a crusher machine (Mainland, Hunan, China), which was then sifted 100 µm by a laboratory test sieve (Endecotts, London, England). PLA was bought under the trade name polylactide resin 3052D from nature works LLC (Minnetonka, MN, USA). It has a range of melting points from 170 to 190 °C, a density of 1.24 g/cm^3,^ and a molecular weight of 93.500 g/mol. Short MWCNTs was purchased from US Research Nanomaterials, Inc. (3302 Twig Leaf LN, Houston, TX 77084, USA) with > 95% purity, outside diameter (OD) < 7 nm, inside diameter of 2 to 5 nm, 0.5 to 2 µm length and density of 0.27 g/cm^3^. A total of 50 g of each sample was prepared for blending. The OPEFB/PLA/MWCNTs composites were fabricated by mixing 6%, 10%, 14%, 18%, and 22% mass percentages of MWCNTs with OPEFB fiber and PLA at a fixed mass ratio of 3:7 as presented in Table 1. Weight percentages greater than 5 were chosen to avoid the marginal effect of MWCNTs on the dielectric properties because lower filler contents could be completely dispersed in the OPEFB/PLA matrix. However, MWCNTs loadings greater than 22 wt.% could lead to formation agglomerates. These agglomerates harm the dielectric and mechanical properties of a material. The PLA was oven-dried at 60 °C for 2 h. The oven-dried PLA was melted in Brabender Internal Mixer for 2 min at 160 ºC with 50 rpm of rotor speed. Then, the OPEFB fiber and MWCNTs powders were added and continued blending for another 12 min. The composites samples were compressed separately to a thickness of 7 mm, (as illustrated in Figure 1) using a hydraulic pressing machine (Fred S. Carver part No.:973110A) in rectangular sample holders at 4 tons, to eliminate air gaps inside the sample likely to influence the results. A total of 20 g of the OPEFB fiber was mixed with polyvinyl chloride (PVC) to make the powders easy to compress into rectangular shapes via hydraulic press machine for complex permittivity measurement.

### 2.2. Dielectric Properties Measurement

**Figure 2:**
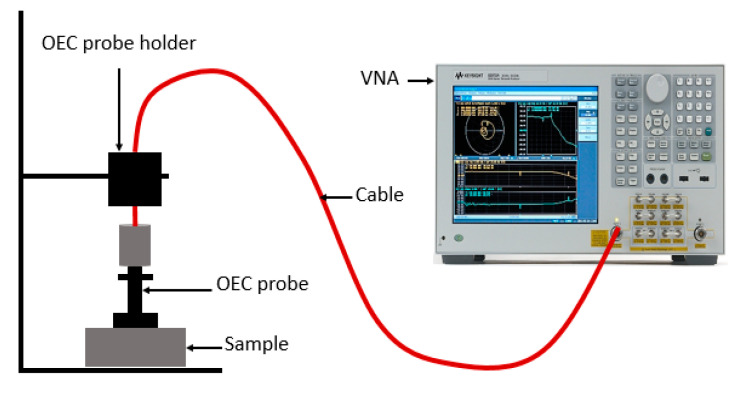
Complex permittivity measurement using open-ended coaxial (OEC).

The measurements for the complex permittivity of the composites were carried out using the open-ended coaxial (OEC) probe 85070B. The probe was connected to an Agilent N5230A PNA-L Vector Network Analyser (VNA, Agilent Technologies, CA, USA) via a high-precision coaxial test cable [19]. The OEC probe is particularly suited for the measurements of complex permittivity of liquid. However, it can be used for solid but special attention should be paid concerning the flatness of the samples and the contact between the sample and the probe. It works from 300 MHz to 20 GHz. A standard one-port, short-air–water calibration was performed and a reference standard material (polytetrafluoroethylene) was characterized to validate the accuracy of the calibration. As illustrated in Figure 2, the OEC probe was then firmly positioned on the flat surface of the samples to determine complex permittivity using the software installed on the VNA. All the measurements were performed at a frequency range of 8–12 GHz.

### 2.3. Measurement of Scattering Parameters (S-Parameters)

Scattering parameters, also known as reflection (S11) and transmission (S21) coefficients, are transmission line parameters often associated with reflection and transmission of electromagnetic waves in microwave networks. The (S21) may define the attenuation characteristics of a material for microwave absorption. The S-parameters were measured using a microstrip line with dimensions of (5 × 6) cm^2^, connected to an HP8720B VNA through the two ports configuration. The VNA’s accuracy depends on the calibration quality standards. The VNA was calibrated at the end of both coaxial cables by implementing a two-port calibration procedure. The Agilent Open-Short-Load Calibration Modul (N4691-60004) automatically measured open, short, and load standards used in the calibration. The measurements were performed at frequency ranged from 1 to 12 GHz. The composites to be measured were covered with a copper foil in other to avoid dispersion of radiation excited through the composite. Firm contact was ensured between the composite base and the microstrip line to avoid air gab during the measurement.

**Figure 3:**
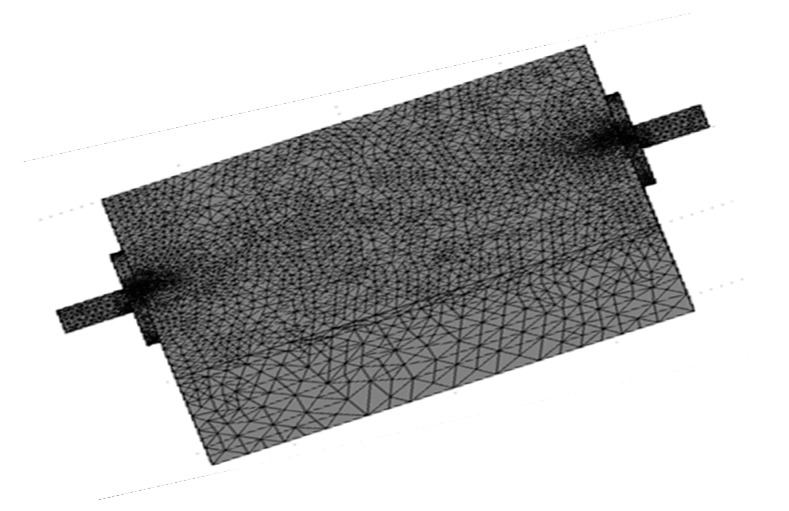
Mesh of microstrip for finite element method (FEM) simulation.

In this study, the finite element method (FEM) was used to model a microstrip for microwave characterization. The frequency and microstrip dimension used was specifically chosen to have a single ${\mathrm{TE}}_{10}$ wave propagating through the microstrip. The simulation was implemented using the COMSOL Multiphysics ^®^ software package, version 3.5. The simulations were based on the model geometry of a microstrip consisting of a dielectric (RT duroid 5880 of complex permittivity of (2.2-j*0.00088)) substrate 6.0 cm long, 5.0 cm wide and 0.15 cm thick and with a signal line (width = 1.5 mm, length = 6.0 cm) etched on the surface of the substrate along the wider side. Before the simulation process, a few steps were required: (a) creating the geometry, (b) defining physical parameters and boundary conditions, (c) meshing the geometry (Figure 3), (d) solving the model geometry, and (e) obtaining the solution. The samples’ measured complex permittivity values were used as inputs for calculations within a 1–12 GHz frequency range.

### 2.4. Characterization

X-ray diffraction (XRD) measurement was performed through a Shimadzu XRD 600 diffractometer (Tokyo, Japan) with a nickel-filtered Cu-Kα (*α* = 0.1542 nm) beam conducted at 30 kV voltage and 30 mA current. The composites were scanned at a scanning rate of 2°/min at 25 °C within a 2-theta range of 10° to 80°. Fourier transforms infrared (FTIR) analysis was carried out through the Perkin Elmer Model 100 series (Waltham, MA, USA). The samples were registered in the wavenumber ranging from 400 to 4000 cm^−1^. Transmission electron microscopy (TEM) of the MWCNTs was performed using Joel 2010F, TEM Akishima, Tokyo, Japan. Field Emission scanning electron microscopy (FE-SEM) measurement was carried out using FE-SEM (Nova NanoSEM 230, FEI Holland) at a fixed voltage of 10 kV.

## 3. Results and Discussion

### 3.1. X-Ray Diffraction (XRD) Analysis

**Figure 4:**
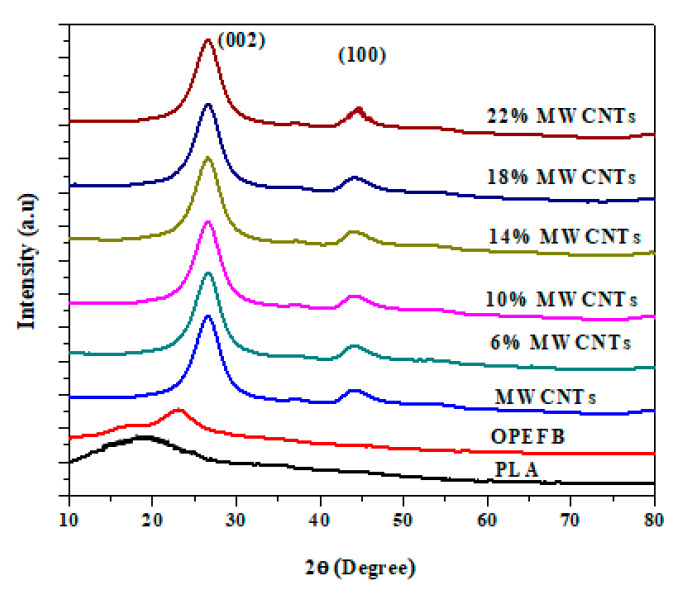
XRD pattern of OPEFB, PLA, MWCNTs, and OPEFB/PLA/MWCNTs composites with different concentrations of MWCNTs.

The OPEFB, PLA, MWCNTs, and OPEFB/PLA/MWCNTs composites were characterized to study the effect of MWCNTs on the OPEFB-PLA composites crystallinity. As presented in Figure 4, the XRD patterns of pure PLA showed a wide diffraction peak of approximately 2θ ≈ 18°. The PLA showed no characteristic peak and indicated that PLA has an amorphous structure [20]. The OPEFB also exhibits peaks at 2θ ≈ 16° and 2θ ≈ 24° indicating amorphous and crystalline regions [21], Cellulosic natural fibers contain both crystalline (ordered) and amorphous (disordered). Thus, the existence of dual features in the fiber is consistent with the presence of ordered and disordered regions [22]. The broad peaks of OPEFB suggest a carbon-based and cellulose type material [12]. The MWCNTs XRD pattern displays a wide diffraction peak at 2θ ≈ 26.2° that corresponds to (002) and a small peak at 2θ ≈ 44.2° corresponding to (100) indicating the crystalline structure of MWCNTs [3]. Moreover, the OPEFB/PLA/MWCNTs nanocomposites showed characteristic diffraction peaks at 2θ ≈ 26.2° and 2θ ≈ 44.2°. The high diffraction peak at 26.4° may be due to the MWCNT stacking layers with the disorder (002) [23].

### 3.2. Fourier Transform Infrared (FTIR) Analysis

**Figure 5:**
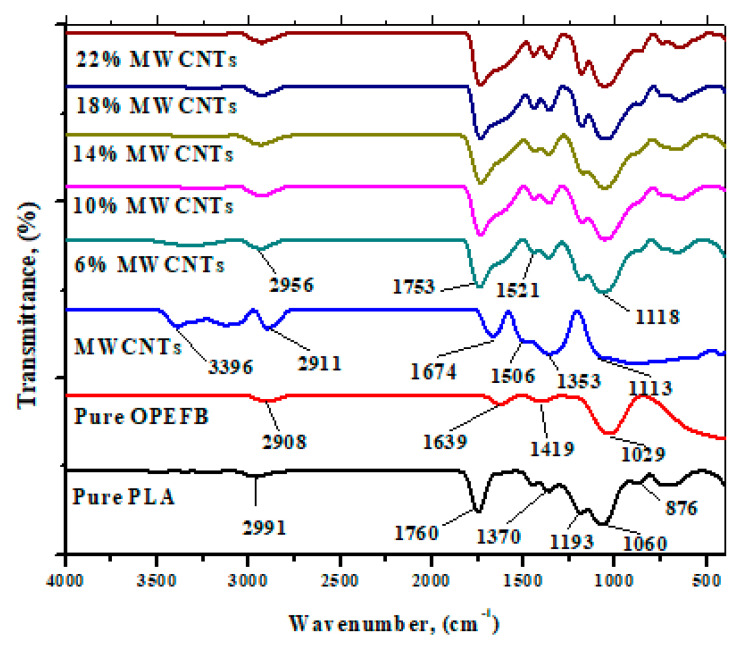
FTIR spectra of OPEFB, PLA, and OPEFB/PLA/MWCNTs composites.

FTIR spectra of OPEFB, PLA, MWCNTs, and OPEFB/PLA/MWCNTs composites with wavenumber ranging from 400 to 4000 cm^−1^ are depicted in Figure 5. The spectrum of MWCNTs exhibits characteristic peaks at 3396 and 2911 cm^−1^ represents –OH and C=O stretching vibrations of carboxylic groups, respectively. The remaining characteristic vibrations that appear as impurities at 1674, 1506, 1353, and 1113 cm^−1^ reflect the aliphatic –CH, C–O, –C−O−H, –OH bending or –C−O−C stretching vibration. In the OPEFB FTIR spectrum, broadband is observed at 2908 and 1760 cm^−1^ respectively, which represents –OH stretching vibration of cellulose, hemicellulose, and lignin. It is evident that with the addition of MWCNTs in the OPEFB/PLA matrix, the characteristic vibrations were slightly towards the higher wavenumber and broader than their samples.

### 3.3. TEM and FE-SEM Analysis

**Figure 6:**
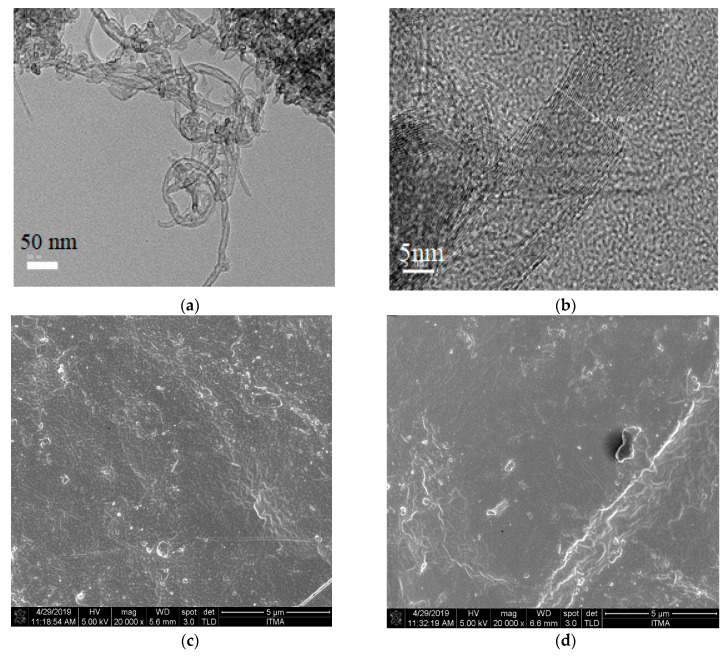
TEM image of (**a**,**b**) MWCNTs and FE-SEM image of (**c**) OPEFB/PLA/6% MWCNTs; (**d**) OPEFB/PLA/22% MWCNTs.

The TEM and FE-SEM images in Figure 6 showed the morphology of WMCNTs and OPEFB/PLA/MWCNTs nanocomposites. Figure 6a,b shows the TEM of the MWCNTs with agglomerates’ average size of around 13.7 nm. Figure 6c,d display the FESEM of OPEFB/PLA/6 wt.% MWCNTs and OPEFB/PLA/22 wt.% MWCNTs nanocomposites. The MWCNTs bind strongly to the OPEFB/PLA matrix surface and tips due to the effectiveness of the Brabender mixer as can be seen in the FESEM images. The uniform dispersion of hybrid filler particles in polymer systems leads to the creation of a co-supportive network that is likely to strengthen composite electrical conductivity. Nanocomposite’s network structure with a high available surface area is expected to contribute to various interfacial polarization resulting in the weakening of the EM waves [6].

### 3.4. Dielectric Properties Analysis

**Figure 7:**
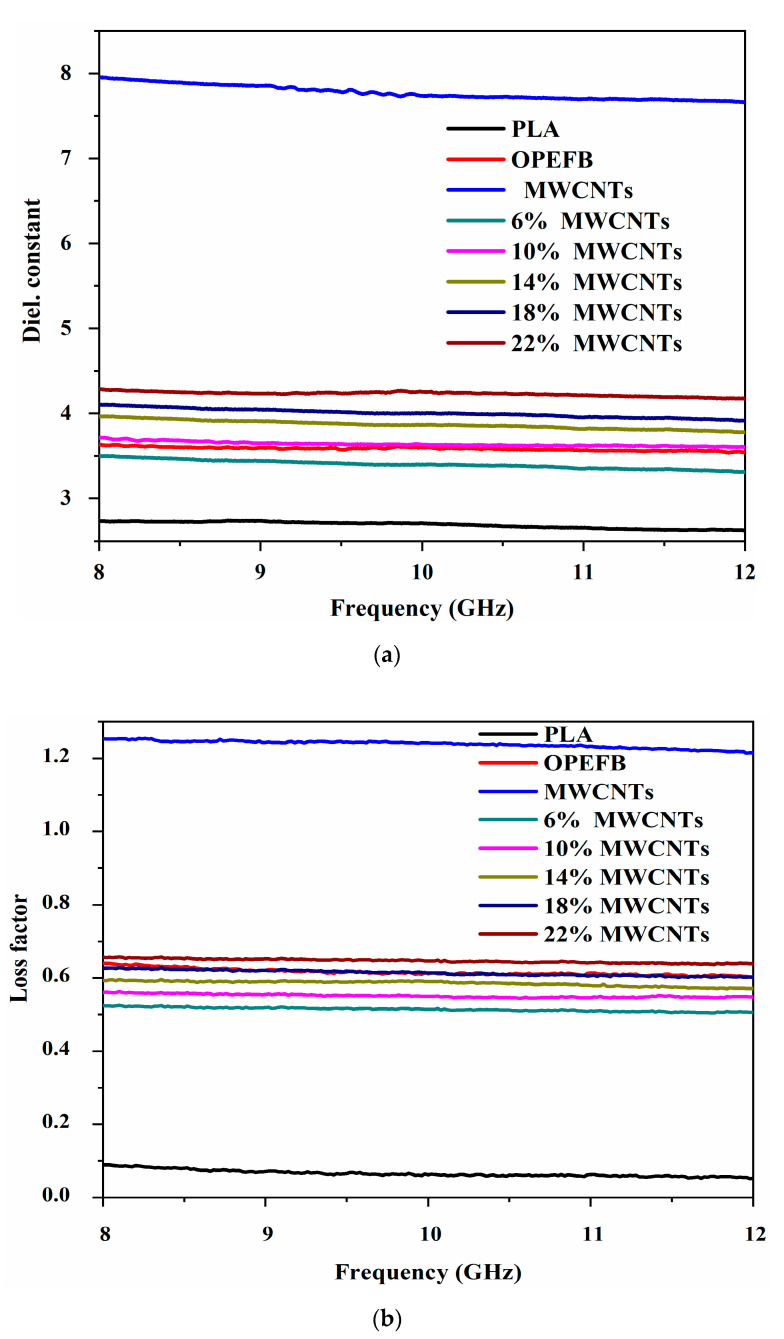
Variation in: (**a**) dielectric constant (ԑ’); (**b**) loss factor (ԑ”) of OPEFB/PLA/MWCNTs.

To analyze the dielectric properties of the MWCNTs, compacted samples with a thickness of 7 mm were characterized for their dielectric constant ($\varepsilon^{\prime }$) and loss factor ($\varepsilon^{\prime \prime }$) magnitudes. Figure 7 displays the complex permittivity of the OPEFB fibers and MWCNTs and the frequency variation. In general, $\varepsilon^{\prime }$ and $\varepsilon^{\prime \prime }$ of both OPEFB fiber and MWCNTs decreased with frequency increase, as described by the Maxwell–Wenger polarization model [24]. Both $\varepsilon^{\prime }$. and $\varepsilon^{\prime \prime }$ values demonstrated instability in their profiles due to impedance mismatch between the OEC input impedance and the surface impedance of the compacted samples resulting from surface imperfections and cracks. The MWCNTs had higher $\varepsilon^{\prime }$ and $\varepsilon^{\prime \prime }$ values in the 8–12 GHz range than the OPEFB fiber. The OPEFB fiber and MWCNTs $\varepsilon^{\prime }$ values were 3.59 and 7.77 while the $\varepsilon^{\prime \prime }$ values were 0.64 and 1.24 respectively. The improved complex permittivity of OPEFB/PLA/MWCNT composites is attributed to the incorporation of MWCNTs that can form more interfaces between the composites and thus improve polarization [25].

The effect of the 6–22 wt.% MWCNTs filler on the dielectric properties of OPEFB/PLA/MWCNTs composites was studied, and the variation in $\varepsilon^{\prime }$ and $\varepsilon^{\prime \prime }$ values in the range of 8–12 GHz is shown in Figure 7. It has been reported that the quality of conductive fillers distributed in a polymer matrix will play a significant role in the entire sample’s dielectric behaviors [26]. PLA polymer has low permittivity because of the small amount of macromolecular polarization. Adding fillers to the polymer can improve the matrix’s reduced permittivity [12] because the polarization of the filler and filler/polymer interfaces (interfacial polarization) can significantly contribute to the composite’s overall polarisation. The values of $\varepsilon^{\prime }$ and $\varepsilon^{\prime \prime }$ in the 8–12 GHz range decreased with frequency for the 6–22 wt.% composites. The complex permittivity values for the composites depend primarily on the contribution of interfacial, orientation, and electronic and atomic polarization in the material, due to the differences in the polarization or conductivity of the matrix and the filler. The results show that the complex permittivity of OPEFB/PLA composites can be significantly increased by the addition of MWCNTs.

### 3.5. Microwave-Absorbing Properties

The absorbing properties of the OPEFB/PLA/MWCNTs composites were obtained from the calculated absorption loss ($SE_{A}$) using the measured transmission (|S_21_|) and reflection (|S_11_|) coefficients determined through VNA. The $SE_{A}$ magnitudes were obtained via the expression given by Equation (1) [27].

(1) $$SE_{21\;\;}=10\log\left(1-{\left|S_{11}\right|}^{2}\right)/{\left|S_{21}\right|}^{2})$$

**Figure 8:**
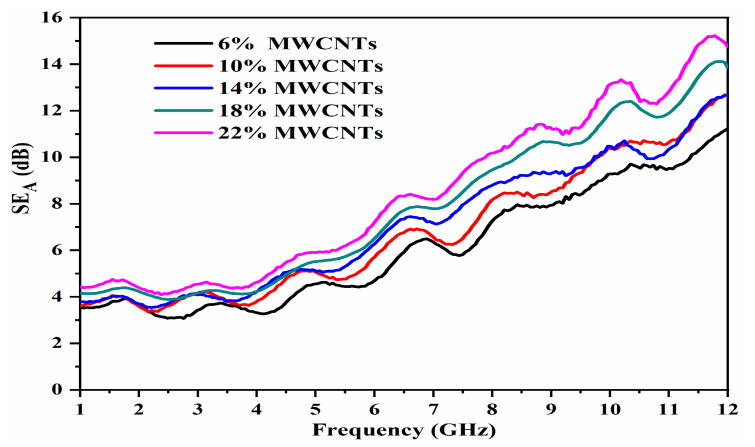
Variation in absorption loss for the OPEFB/PLA/MWCNT nanocomposites.

The SE_A_ values of the composites increased across the 1–12 GHz range as presented in Figure 8. The composites’ SE_A_ values increased from 7.4 at 8 GHz to 15.2 dB at 11.75 GHz frequency. In these dipoles, electron motion hysteresis under an alternating electromagnetic field generated additional polarization relaxation processes that were important in improving microwave absorption properties [27]. There is no substantial variation in the values of SE_A_, considering 6–22 wt.% composites. This small variation is probably due to measurement uncertainties during the permittivity measurements. These findings demonstrate that the OPEFB/PLA/MWCNTs are capable of significant microwave absorbing and should act as a cheaper and more effective option for applications throughout the investigated frequency range. Table 2 shows the variation in $SE_{A}$ for the OPEFB/PLA/MWCNTs composites.

**Figure 9:**
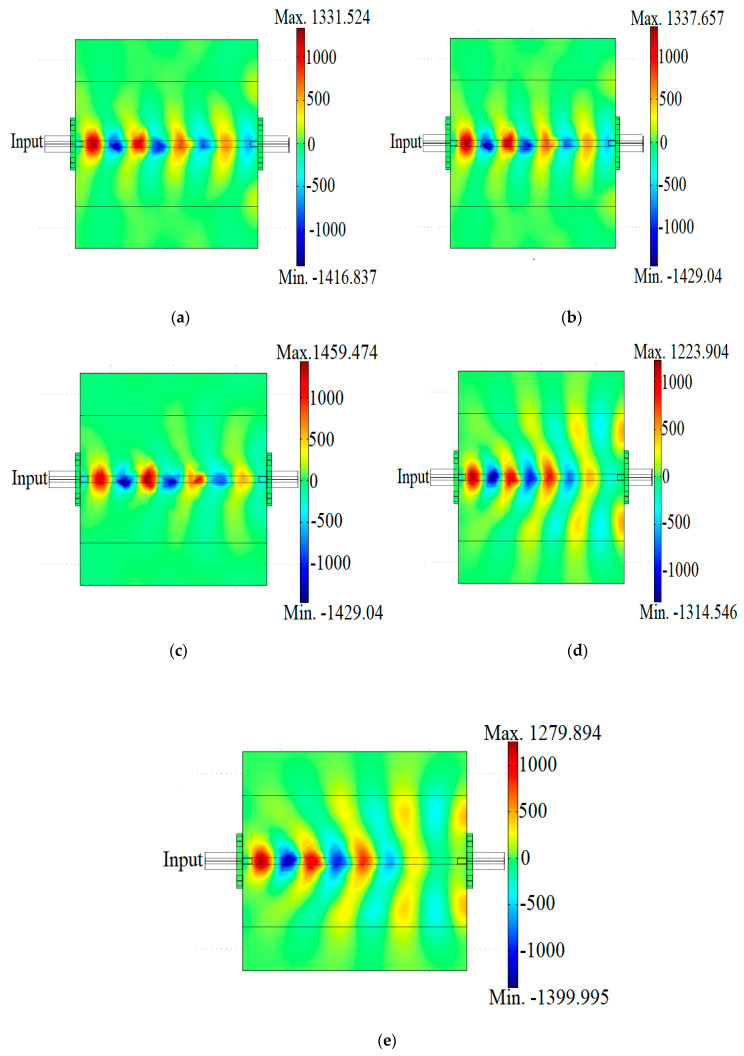
Electric field distribution patterns for OPEFB/PLA/MWCNT nanocomposites: (**a**) 6%; (**b**) 10%; (**c**) 14%; (**d**) 18%; (**e**) 22% MWCNTs.

FEM simulation results of the electric field (V/m) distribution at the X-band frequency of the microstrip covered with OPEFB/PLA/MWCNTs composites is presented in Figure 9. The results revealed that the pattern of radiation from the electric field distributed through the samples depends on the permittivity values. The red color represents the electric field while the blue color represents absorption loss. The electromagnetic wave propagates from the input to output, the higher the $\varepsilon^{\prime \prime }$ the less energy at the output due to reflection at the input and absorption at the output. It is observed that as the MWCNTs loadings increased the weaker the blue color (Figure 9a–e) which implies more absorption. As anticipated the higher loss material has higher absorption and thus lower electromagnetic wave transmission through the sample [19].

## 4. Conclusions

In this study, OPEFB/PLA/MWCNTs nanocomposites were successfully fabricated with 6–22 wt.% MWCNTs via the melt-blend technique. The composites were characterized for complex permittivity using the coaxial probe technique at 8 to 12 GHz frequency range. The ε′ and ε″ values of all the composites increased with increase in MWCNTs filler content. The ε″, crucial for microwave absorption properties, increased from 0.51 to 0.65 when the MWCNTs filler was increased from 6–22 wt.%. Furthermore, to obtain the absorption loss ($SE_{A}$) of the OPEFB/PLA/MWCNTs composites, the Scattering parameters (*S_11_* and *S_21_*) were measured via the microstrip line technique. The results show that with the incorporation of MWCNTs filler, the complex permittivity and absorption properties of the nanocomposites improved. The highest microwave absorbing property (SE_A_) 15.2 dB was obtained for the 22 wt.% nanocomposites at 11.75 GHz. The simulated absorption properties showed by the composites proved their capacity to attenuate the X-band frequency microwave. The OPEFB/PLA/MWCNTs nanocomposites are biodegradable, easy to synthesize, cheap, and effective for microwave absorbing application in the X-band frequency range.

## Figures and Tables

**Table 1:** Composition of oil palm empty fruit bunch (OPEFB) fiber/polylactic acid (PLA)-based composites filled with 6–22 wt.% multi-walled carbon nanotubes (MWCNTs) composites.

OPEFB		PLA		MWCNTs		Total Mass (g)
Weight (%)	Mass (g)	Weight (%)	Mass (g)	Weight (%)	Mass (g)	
28.2	14.1	65.8	32.9	6	3	50.0
27.0	13.5	63.0	31.5	10	5	50.0
25.8	12.9	60.2	30.1	14	7	50.0
24.6	12.3	57.4	38.7	18	9	50.0
23.4	11.7	54.6	27.3	22	11	50.0

**Table 2:** Variation in absorption loss ($SE_{A}$) for the OPEFB/PLA/MWCNTs composites.

MWCNTs (wt.%)	8 GHz	9.5 GHz	10.5 GHz	11.7 GHz
6	7.4	8.4	9.5	10.9
10	8.2	9.3	10.6	12.4
14	8.8	9.5	10.1	12.5
18	9.5	10.6	12.1	14.1
22	10.2	11.3	12.3	15.2
